# On the Limits of Scanning Thermal Microscopy of Ultrathin Films

**DOI:** 10.3390/ma13030518

**Published:** 2020-01-22

**Authors:** Christoph Metzke, Werner Frammelsberger, Jonas Weber, Fabian Kühnel, Kaichen Zhu, Mario Lanza, Günther Benstetter

**Affiliations:** 1 Department of Electrical Engineering and Media Technology, Deggendorf Institute of Technology, Dieter-Görlitz-Platz 1, 94469 Deggendorf, Germany; christoph.metzke@th-deg.de (C.M.); jonas.weber@th-deg.de (J.W.); fabian.kuehnel@stud.th-deg.de (F.K.); 2 Department of Mechanical Engineering and Mechatronics, Deggendorf Institute of Technology, Dieter-Görlitz-Platz 1, 94469 Deggendorf, Germany; werner.frammelsberger@th-deg.de; 3 Institute of Functional Nano and Soft Materials, Collaborative Innovation Center of Suzhou Nanoscience & Technology, Soochow University, 199 Ren-Ai Road, Suzhou 215123, China; Kczhu1995@126.com (K.Z.); mlanza@suda.edu.cn (M.L.); 4 Department of Applied Physics, University of Barcelona, Martí i Franquès 1, 08028 Barcelona, Spain

**Keywords:** scanning thermal microscopy, SThM, Atomic force microscopy, AFM, boron nitride, h-BN, topography influences, ultrathin films, copper iodide, CuI

## Abstract

Heat transfer processes in micro- and nanoscale devices have become more and more important during the last decades. Scanning thermal microscopy (SThM) is an atomic force microscopy (AFM) based method for analyzing local thermal conductivities of layers with thicknesses in the range of several nm to µm. In this work, we investigate ultrathin films of hexagonal boron nitride (h-BN), copper iodide in zincblende structure (γ-CuI) and some test sample structures fabricated of silicon (Si) and silicon dioxide (SiO_2_) using SThM. Specifically, we analyze and discuss the influence of the sample topography, the touching angle between probe tip and sample, and the probe tip temperature on the acquired results. In essence, our findings indicate that SThM measurements include artefacts that are not associated with the thermal properties of the film under investigation. We discuss possible ways of influence, as well as the magnitudes involved. Furthermore, we suggest necessary measuring conditions that make qualitative SThM measurements of ultrathin films of h-BN with thicknesses at or below 23 nm possible.

## 1. Introduction

Miniaturization is an economically driven continuous process that applies to Integrated Circuits (ICs) as well as for complete microsystems. In the following the term microsystem (MS) is used for diverse miniaturized systems with feature sizes from the nanometer to the micrometer range that include ICs. With decreasing feature sizes device densities and current densities have increased [1,2] and Joule heating has become increasingly critical. Today, in defect-free, metallic carbon nanotubes, it is possible to reach current densities with values of 10^10^ Acm^−2^, which is equivalent to 7 GA current for standard household wires [3]. Household wires would immediately melt, but MSs carrying structures with similar current densities are still in service. The heat transfer capabilities of these thin films are critical, as MS typically constitute of a stack of diverse thin film layers. In future, the availability of suitable new thin film materials is an important issue for further progress, but the associated local thermal characterization is at least equally important. Unfortunately, the thermal properties of thin films substantially differ from the corresponding bulk material [4,5,6,7] and are much harder to acquire. The present work focuses on the metrology of local thermal properties of thin film materials for MSs and the associated restrictions and artefacts. 

The difference between thermal properties of thin films and corresponding bulk material stems from several atomic and molecular effects [4,5,6,7] and from the fact that a thin layer constitutes more or less a surface and interface effects become dominant over bulk effects. A further complication is that local anisotropies and thickness fluctuations increasingly affect the thermal properties with decreasing film thickness. Furthermore, thermal conductivities in thin films strongly depend on the grain size. Fladischer et al., who investigated 500 nm thin, chemical vapor deposition (CVD) grown tungsten films, showed this. After a long heat treatment (4 cycles of ∼20 min. at 425 °C) the thermal conductivity increased from 153 Wm^−1^ K^−1^ to 155 Wm^−1^ K^−1^, caused by a growth of the grain sizes [8]. The cross and in-plane thermal conductivities in thin films can also differ in various magnitudes. It can be necessary to consider both the diffusive in-plane heat flux and the ballistic Stefan-Boltzmann like cross-plane heat flux, depending on the film thickness and the phonon mean-free path. Heiderhoff et al. studied this transition of heat transport, specifically in non-crystalline TiO_2_ films with decreasing film thicknesses [9]. Very local characterization techniques with a resolution in the nanometer range are needed to investigate such effects. Scanning thermal microscopy (SThM) is an appropriate method for characterizing the local thermal properties of thin films [10,11,12,13]. Earlier observations indicated [14,15,16] that the SThM results may be affected by artefacts that are associated with the sample topography and the measurement system itself. Therefore, in this work, we assess the influence of sample topography, tip sample contact angle, and probe temperature on the thermal SThM signal and evaluate and discuss the impact on the measurement results with respect to the thermal conductivity of the film under investigation.

SThM thermal images are likely to be influenced by the sample’s topography, which has been explained in literature in recent years. Hammiche et al. [12] found out that, even for homogeneous samples, variations of the thermal contact area between tip and sample lead to an alternating heat flow. His research was conducted while using homogeneous polystrene. Lehermeier [14] studied the thermal properties of γ-CuI thin films. His thermal SThM images showed a higher local thermal conductivity at grain boundaries when compared to the conductivity inside a grain. He could not exclude a significant topography influence on SThM measurements. Furthermore, Majumdar et al. [11] and Sadehi et al. [17] discovered topography-related artefacts in their investigations. Shi et al. [18] made SThM measurements of carbon nanotubes with different applied heating voltages to eliminate the topography influence. Ruiz et al. [19] showed that after proper calibration SThM measurements are suitable for measuring absolute values of the thermal conductivity of diamond-like nanocomposite materials but Price et al. [13] critically questioned his results. It would not be routinely possible to make absolute measurements of local thermal conductivities, since the thermal signal was convoluted with the surface roughness. Sharp changes in relief would affect the contact area and, thus, the heat exchange between tip and sample, which would falsify the thermal image. Martinek et al. [20] used nethods, such as neural network analysis and three-dimensional (3D) finite element modelling, to reduce this topography influence. An excellent overview about the fundamentals and applications of SThM is given in the review of Gomès et al. [21].

This work considers the topography influence (Section 4.1), the temperature dependency (Section 4.2), and the angle dependency (Section 4.3) to further study artificial effects in SThM measurements. We investigate ultrathin films of hexagonal boron nitride (h-BN), thin films of copper iodide in zincblende structure (γ-CuI), and a test sample of silicon dioxide (SiO_2_) arranged in steps and circles on top of bulk silicon (Si) by means of SThM. The film thickness of the h-BN sample ranges from approximately 5 nm to 23 nm and, thus, approaches thickness values close to so called 2D materials. Generally speaking, 2D materials are crystalline materials that only contain one atomic layer, so there is virtually no third dimension. With their special mechanical, electrical, and thermal properties, such as significant higher thermal conductivities and mechanical strength when compared to the bulk materials [22], they are ideal candidates for the use in MSs. Promising candidates are 2D h-BN, investigated here, graphene and 2D MoS_2_ [23,24]. Other promising materials for future SThM investigations are diamond like films [25,26]. In recent years, research activities on 2D materials, including the evaluation and development of associated characterization techniques, have enormously increased as the film thicknesses in MSs approaches atomic level. This work provides another important contribution to the development and improvement of suitable investigation methods, on the one hand, and to the thermal characterization of ultrathin h-BN films, on the other hand. 

## 2. Materials and Methods

### 2.1. Atomic Force Microscopy (AFM) and Scanning Thermal Microscopy (SThM)

In this work, we employ the following measuring equipment, which are explained in more detail hereafter:

- AFM system: Bruker Dimension Icon [27]; operating software: NanoScope 9.1
- Thermal system: Bruker Vita Module [28] consisting of a power supply, a controller, a calibration box, a variable resistor box, the nano-TA calibration sample, and the software Bruker VITA studio v3.4
- Thermal probe: SThM probe Bruker VITA-DM-NANOTA-200 [29]; The thermal probe is made from crystalline silicon (Si) and can be heated repeatedly and reliably up to temperatures of 350 °C, according to manufacturer information. In the following, we use the term “probe” for the whole thermal probe (as it can be purchased), and the term “tip” only for the sharp area on the front side of the probe, which is directly touching the surface of the samples.

AFM is a method for characterizing surfaces according to the topographical, mechanical, and electrical [30] properties on a nanometre scale. The sample is scanned with a predefined number of lines and readings per line, which results in an image line by line. It utilizes different parameter interactions between the probe and the investigated surface, such as the force, current, or voltage. One of the main parts is a microfabricated probe with a sharp tip. Tip radii are in the small nm range (depending on the AFM method employed), e.g., Bruker RESP-20 with a tip radius of 8 nm to 12 nm and a tip height of 10 nm to 15 nm [31]. This probe is mounted on a piezoelectric actuator on whose backside a laser beam is focused. The backside of the probe reflects the laser beam and directed to a photo detector. By this means that it is possible to determine the deflection of the probe and, hence, the topography of the sample. The three fundamental modes of AFM are the contact mode (Cont-AFM), the non-contact mode (NC-AFM) and the intermittent-contact mode (IC-AFM), which combines Cont-AFM and NC-AFM. Typically, the force between the tip and sample in Cont-AFM is in the range of 10 nN to 100 nN [32,33].

SThM is a method for qualitatively mapping local thermal conductivities [34]. SThM measurements create two images of the same position simultaneously, one of the topography and one of the corresponding thermal properties. It is used in an AFM system together with an additional thermal system. The AFM operates in contact mode, in which the tip scans over a sample directly touching the surface. SThM utilizes the force interaction between the probe and sample to produce topography images and variations of the electrical resistance within the probe to record thermal images. The thermal system is responsible for set up, calibration procedure, and thermal measurement. Both of the systems combined provide a local thermal signal that is assigned simultaneously to the corresponding topography and the lateral tip position. For this, a thermal resistive probe is heated with a constant heating power. The temperature of the probe depends on the heat exchange between tip and sample and, therefore, on the sample’s local thermal conductivity. If the local thermal conductivity of the sample is high, more heat can spread into the sample and so the temperature of the tip (and consequently the temperature of the probe) decreases and vice versa. A complex extension of the SThM method makes it possible to quantitatively map local thermal conductivities [35,36]. However, this is not part of the present work.

**Figure 1:**
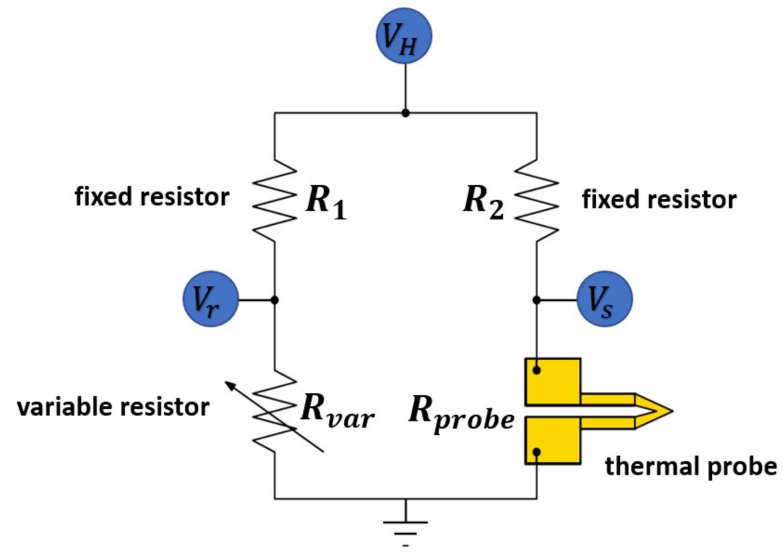
Wheatstone bridge in Scanning thermal microscopy (SThM) measurement. The electrical resistors $R_{1}$ and $R_{2}$ are fixed. $R_{probe}$ is the temperature dependent electrical resistance of the thermal probe. Before the measuring process the Wheatstone bridge is balanced to $V_{out}{\;=\;V}_{s}\;-{\;V}_{r}\;=\;0$ by means of adjusting $R_{var}$ (based on [14]).

At the beginning of the measuring process, the Wheatstone bridge of the thermal system (Figure 1) is balanced by manually adjusting the variable resistor $R_{var}$ in the variable resistor box. During this set-up process, the scanning process is disabled and the tip position is fixed. The thermal signal, represented by $V_{out}{\;=\;V}_{s}\;-V_{r}\;$, is transferred to the thermal system and it can be set to zero there. After set-up the scanning process can be started. While the probe is scanning over the surface the local thermal conductivities vary. At a position with a high thermal conductivity, the probe temperature decreases as more heat spreads into the sample. As a result, the temperature dependent electrical resistance of the probe, $R_{probe}$, decreases and the Wheatstone bridge becomes unbalanced, imposing a negative thermal signal $V_{out}{\;=\;V}_{s}\;-{\;V}_{r}\;<\;0$. Dark colours in the thermal images that are presented here represent negative signals so that, in our measurements, “black” positions correspond to a high local thermal conductivity. The influence of thermal radiation and convection on the thermal results may be excluded, because the probe’s temperature is held nearly constant (only varies in the tenth-Kelvin range) by the system during the measurements.

### 2.2. Samples and Materials under Investigation

In this work, we investigate three different samples:

- Test sample TGXYZ02 [37]
- Copper iodide in zincblende structure (γ-CuI)
- Hexagonal boron nitride (h-BN).

**Figure 2:**
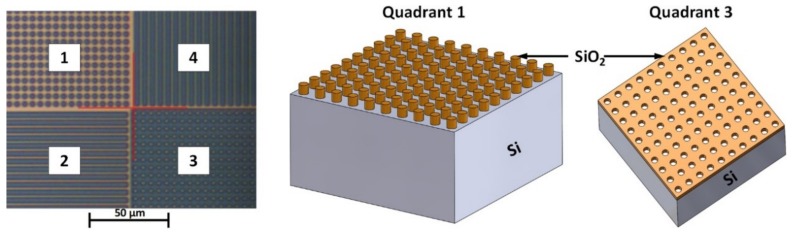
Active area of the test samples. Quadrant 1: raised circles. Quadrant 2: horizontal raised steps. Quadrant 3: submerged circles. Quadrant 4: vertical raised steps.

The test sample TGXYZ02 [37] was fabricated by the company MikroMasch^®^ and it contains a step and circle structure (SC-structure). This SC-structure is made of silicon dioxide (SiO_2_) on top of a bulk silicon substrate. According to the manufacturer, the SiO_2_ film is 96 nm ± 3% high and the pitch is 5 µm ± 0.1 µm. Overall, the chip has dimensions of 5 mm × 5 mm × 0.3 mm [37]. The active area is 1 mm × 1 mm and it contains a test pattern, as illustrated in Figure 2. The test structures will not be thermally damaged by the heated tip during the SThM measurements, because bulk Si and SiO_2_ possess melting temperatures (1410 °C and 1710 °C, respectively) far beyond the maximum tip temperature of 200 °C. The thermal conductivity of the SC-structure is significantly lower (bulk SiO_2_: 1.38 Wm^−1^ K^−1^) than the thermal conductivity of the Si substrate (bulk Si: 163 Wm^−1^ K^−1^).

The second film under investigation consists of copper iodide in zincblende structure (γ-CuI). CuI is a binary metal halide semiconductor, which is transparent for visible light. Its band gap in zincblende structure (γ-CuI) is approximately 3.1 eV. Recently, excellent thermal and electrical properties of CuI have been reported, which make it a promising candidate for improved thermoelectric [38,39] and optoelectronic [40] applications. In this work, we investigate thin films of γ-CuI with a thickness of approximately 1 µm on top of an amorphous glass structure. The thermal conductivity of this γ-CuI structure can be estimated to 0.5 Wm^−1^ K^−1^ [38].

The surface of the third sample is fabricated of hexagonal boron nitride (h-BN). In general, it contains 15 to 18 layers of h-BN with a thickness of 0.33 nm to 0.4 nm each above a 300 nm thick SiO_2_ structure. Therefore, we can estimate the general h-BN thickness to 6.075 nm ± 1.125 nm. The bottom substrate is made of Si, with a thickness of approximately 1.5 mm. On the top layer, local rests of PMMA particles may be present, which are residuals from the manufacturing process. However, all of the thermal measurements have been carried out at PMMA-free sites, as confirmed by topographic maps. The useful position investigated in this work (Section 3.2) contains a vertical step with more layers of h-BN caused by the manufacturing process, to make qualitative SThM measurements possible. With a step height of 15.5 nm, we can estimate the h-BN thickness on top of the step to 21.575 nm ± 1.125 nm. Layers of h-BN are electrically insulating and they possess a comparatively high thermal in-plane conductance at the same time. Together with other chemical, mechanical, and electrical properties, h-BN is a promising 2D material for future semiconductor devices in the field of optoelectronics, functional composites, accumulators, or electrically insulating substrates. It seems to have high potential for electrical insulating layers, which can spread heat away from critical structures effectively [41]. The properties of h-BN are explained here in more detail due to its potential. The highly anisotropic thermal conductivity of h-BN is the most important property, as far as this research is concerned. The in-plane thermal conductivity is estimated to be between 100 Wm^−1^ K^−1^ and 2000 Wm^−1^ K^−1^, whereas the cross-plane values fall to only a few Wm^−1^ K^−1^ [42,43,44]. Boron nitride nanosheet (BNNS), another term for 2D h-BN, is a layered material with a graphite like structure [45]. This structure is frequently called the honeycomb-structure due to its hexagonal weave. Nitrogen and boron atoms are regularly arranged and they are held together by covalent bonds. Van der Waals forces cause the attraction between two layers. The lattice mismatch is only 1.7% [46], which makes the h-BN structure similar to graphene. Therefore, it is also possible to combine h-BN layers and graphene layers to a hybrid structure for fully two-dimensional (2D) metal-insulator-semiconductor (MIS) composites [47]. Unlike graphene, h-BN is an electrical insulator with a band gap between 5.2 eV and 5.9 eV. It has low dielectric losses with a permittivity between 2 and 4 [47,48]. The hexagonal structure is stable until a melting point of approximately 2967 °C and, therefore, will not be affected by our SThM measurements (tip temperature < 200 °C). The use of h-BN in semiconductor devices is still at a very early stage and there is a huge variety of other promising 2D materials for MS, such as MoS_2_, WS_2_, MoSe_2_, and WSe_2_ [41]. The first synthesis of BNNS was performed in 1842 by the reaction between boric oxide and potassium cyanide [49]. Today, CVD is the most effective technique for large-scale synthesis of BNNS. Optical microscopy, Raman spectroscopy, Fourier transform infrared spectroscopy (FITR), scanning electron microscopy (SEM), transmission electron microscopy (TEM), and AFM are the most common characterization methods [41].

## 3. Experimental Set-up

### 3.1. Probe Calibration

Before measuring with SThM the calibration of the probe is necessary. That ensures that the applied heating voltage corresponds to the correct probe (and also tip) temperature. After probe calibration, it is possible to set a specific temperature and the software automatically applies the corresponding voltage to the probe. Thus, it is possible to assign the thermal image acquired to the corresponding probe temperature. Calibration is performed with the software VITA Studio v3.4 and the nano-TA calibration sample, which are part of the thermal system [28]. The nano-TA calibration sample contains the following three materials with different melting points $T_{m}$:

- Polycaprolactone (PCL; $T_{m}\;=$ 55 °C)
- Polyethylene (PE; $T_{m}\;=$ 116 °C)
- Polyethylene terephthalate (PET; $T_{m}\;=$ 235 °C)

The calibration procedure was performed at two random positions of each material to increase the accuracy of the calibration procedure. At the beginning of this process, the tip is brought to the sample’s surface by the piezoelectric actuator until it slightly touches the surface. The probe deflection is then set to zero. Currently, the temperature of the probe is increased in a system specific temperature ramp (defined within the thermal system), so that probe, tip, and locally the calibration sample heat up. As a consequence, the sample material expands and thus, the deflection of the probe increases as the z-position of the probe clamping is fixed. After reaching the melting point of the calibration sample material the tip sinks into the surface and hence the deflection of the probe sharply decreases. In contact mode, the AFM records the deflection of the probe, represented in mV, and assigns it to the corresponding heating voltages. As the melting points of the three calibration materials are known the probe temperature can be assigned to the corresponding heating voltages. For the present study, the procedure described yielded the calibration values that are given in Table 1.

Subsequently, the thermal system performs a quadratic fit in order to relate the heating voltage $V_{H}$ to the probe temperature *T*: ${f(x)\;=\;\mathrm{ax}}^{2}\;+\;\mathrm{bx}\;+\;c$. The coefficients, a, b, and c, are calculated while using the values of Table 1. The result is given in Equation (1), with which it was possible to estimate the probe temperature in our investigations:

(1) $$V_{H}\left(T\right)=-8\times 10^{-5}\;\frac{V}{{\left({}^{\circ}C\right)}^{2}}T^{2}+0.045\;\frac{V}{{}^{\circ}C}T+0.673\;V$$

### 3.2. Useful Sample Positions of h-BN Sample

**Figure 3:**
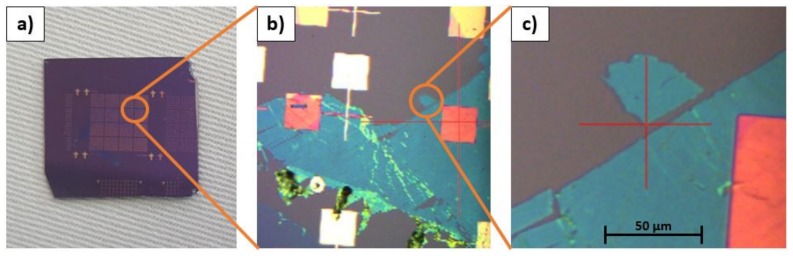
Useful positions on the h-BN sample. (**a**) Entire sample with test structure. (**b**) and (**c**) Zoom into investigated area. We studied positions with different thicknesses of h-BN caused by scrapping off of some h-BN layers during the manufacturing process. The golden test structure was utilized for other measurements and is not relevant for this work.

Before performing our measurements, it was not completely clear whether SThM could be applied to ultrathin films with high in-plane thermal conductivities like h-BN. Similar measurements have not been found in literature to the present time. Therefore, samples with few layer h-BN were investigated. We focused on the thermal contrast at positions in the transition zones between different thicknesses of h-BN, as the system utilized in this work is not able to quantitatively map local thermal conductivities. As our sample shows sites, where a few layers of h-BN were added caused by the manufacturing process (general h-BN thickness: 6.075 nm ± 1.125 nm; h-BN thickness on top of the vertical step: 21.575 nm ± 1.125 nm), it is perfect to qualitatively study the thermal conductivity of few layer h-BN with different thicknesses. Figure 3 exemplarily illustrates a useful position on the h-BN sample. Finding useful positions regarding the γ-CuI and TGXYZ02 samples in this work was not as critical, as the film thicknesses were much higher (>100 nm) and in-plane thermal conductivities much lower (<165 Wm^−1^ K^−1^) when compared to the h-BN sample. Furthermore, the special geometry of TGXYZ02 and the high roughness of γ-CuI (R_q_ of 110 nm, whereas h-BN sample had an R_q_ of 4.15 nm) simplify the search for useful positions.

## 4. Results and Discussion

### 4.1. Demonstration of Topography Influences of SThM Using a γ-CuI Sample

**Figure 4:**
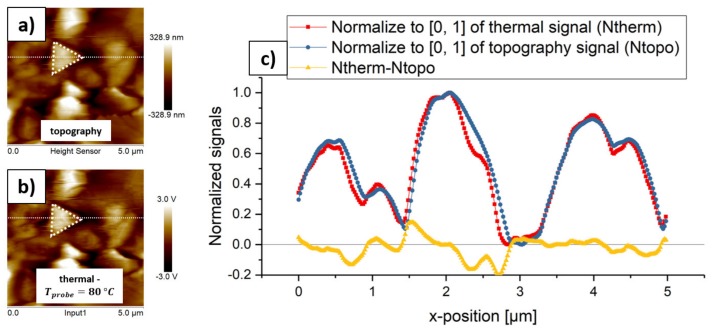
(**a**) Unmodified topography image of a γ-CuI thin film with the corresponding original (as recorded) height scale. (**b**) Unmodified thermal image under a probe temperature of 80 °C with the corresponding original thermal scale. (**c**) Graphical representation of the normalized thermal signal (Ntherm) and normalized topography signal (Ntopo) at the white cut lines of (**a**) and (**b**). Subtracting Ntopo from Ntherm results in the yellow subtraction curve, which is fluctuating around zero.

We performed SThM measurements on γ-CuI samples with a probe temperature of 80 °C to demonstrate the topography influence of SThM. Figure 4a,b illustrate the topography and thermal images. These images show a strong topography influence, as the γ-CuI grains are also visible on the thermal image marked by the white-dotted triangles. The two-dimensional (2D) plots of the normalized thermal and the normalized topography signal at the white cut line look quite similar (compare red squares and blue dots in Figure 4c), highlighting the strong topography influence in this SThM measurement. The subtraction of the normalized topography signal (Figure 4c, blue dots) from the normalized thermal signal (Figure 4c, red squares) results in a curve that fluctuates around zero (Figure 4c, yellow triangles). The mean value of the yellow subtraction curve is −0.02226 (n: 256; median: −0.01036), with a standard deviation of 0.06459. The minimum value is −0.20071 and the maximum value is 0.14675. As the whole surface consists of CuI grains, the local thermal conductivities should nearly be equal on every position, which would result in a subtraction curve similar to the topography signal with an offset on the y-axis. It might also be possible that the course of the thermal signal is influenced by the substrate’s local thermal conductivity at positions, where the thickness of γ-CuI is quite thin, with only a few grains of γ-CuI. However, the strong match of the topography and thermal curve cannot be denied. In summary, the subtraction curve fluctuating around zero is a strong indicator for the topography influence on these SThM-measurements while using γ-CuI. Producing similar curves like Figure 4c at different cut lines results in similar subtraction curves, which also fluctuate around zero with statistical values that are similar to those given above. In the following sections, we discuss the influence of the parameters probe temperature and touching angle in more detail.

### 4.2. Probe Temperature Dependency of the SThM Signal

**Figure 5:**
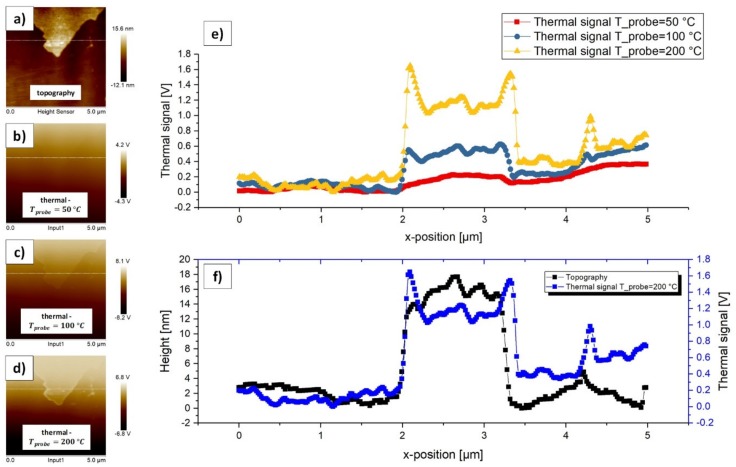
(**a**) Unmodified topography image of an h-BN thin film with the corresponding original (as recorded) height scale containing a step. (**b**) Unmodified thermal image under a probe temperature of 50 °C with the corresponding original thermal scale. (**c**) Unmodified thermal image under a probe temperature of 100 °C with the corresponding original thermal scale. (**d**) Unmodified thermal image under a probe temperature of 200 °C with the corresponding original thermal scale. (**e**) Corresponding 2D plots of the thermal signal at the white cut lines in (**b**), (**c**), and (**d**). The thermal signals were applied with an offset on the y-axis so that the minimum is always zero. (**f**) Comparison of the topography signal at the white cut line in (**a**) with the thermal signal at the white cut line in (**d**), where $T_{\mathrm{probe}}$ is 200 °C.

**Figure 6:**
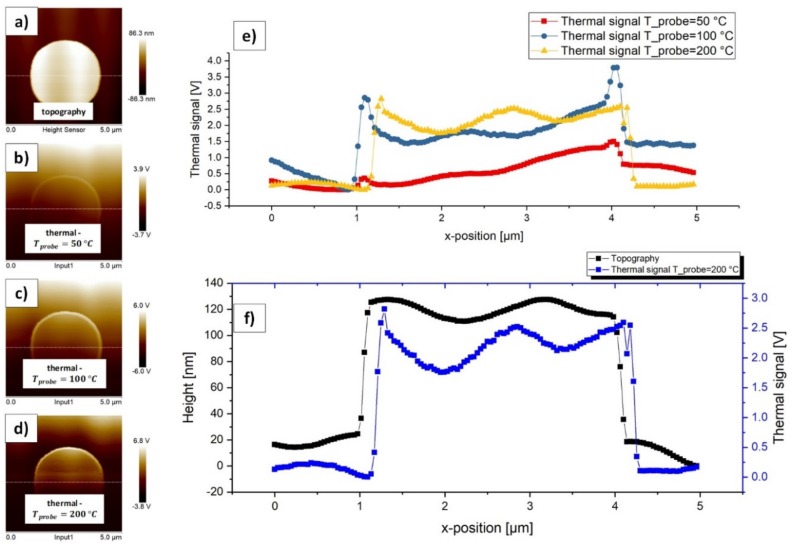
(**a**) Unmodified topography image of the raised circles of the test sample TGXYZ02 (quadrant 1 in Figure 2) with the corresponding original (as recorded) height scale. (**b**) Unmodified thermal image under a probe temperature of 50 °C with the corresponding original thermal scale. (**c**) Unmodified thermal image under a probe temperature of 100 °C with the corresponding original thermal scale. (**d**) Unmodified thermal image under a probe temperature of 200 °C with the corresponding original thermal scale. (**e**) Corresponding two-dimensional (2D) plots of the thermal signal at the white cut lines in (**b**), (**c**) and (**d**). The thermal signals were applied with an offset on the y-axis so that the minimum is always zero. (**f**) Comparison of the topography signal at the white cut line in (**a**) with the thermal signal at the white cut line in (**d**), where $T_{\mathrm{probe}}$ is 200 °C.

The following measurements were performed in order to study the probe temperature dependency of the thermal signal:

1. h-BN on SiO_2_: Measurement positions similar to Figure 3; Probe temperatures: 50 °C, 100 °C, and 200 °C (see Figure 5)
2. TGXYZ02 raised circles: Quadrant 1 in Figure 2; Probe temperatures: 50 °C, 100 °C, and 200 °C (see Figure 6)

We investigated the thermal contrast by means of the following statistic parameters:

- Mean thermal contrast: The thermal signals in each diagram in Figure 5 and Figure 6 were applied with an offset on the y-axis, so that the minimum is always zero. This is permitted, as the thermal signal represented by voltage is a potential unit. Hence, the mean value between the two maximum values (left and right of the step) is defined as “mean thermal contrast”. Those two measuring points define the statistic area and represent the local maxima of the thermal signal on top of the step where the difference quotients of first order change their signs. For the h-BN sample with $T_{\mathrm{probe}}=$ 50 °C (red curve in Figure 5e) there are no corresponding maxima for which reason we use the same statistic area as the one from $T_{\mathrm{probe}}=$ 100 °C.
- Standard deviation of the mean thermal contrast.
- Minimum thermal contrast: Minimum value within the statistic area.
- Median thermal contrast: Median value within the statistic area.
- Maximum thermal contrast: Maximum value within the statistic area.

**Figure 7:**
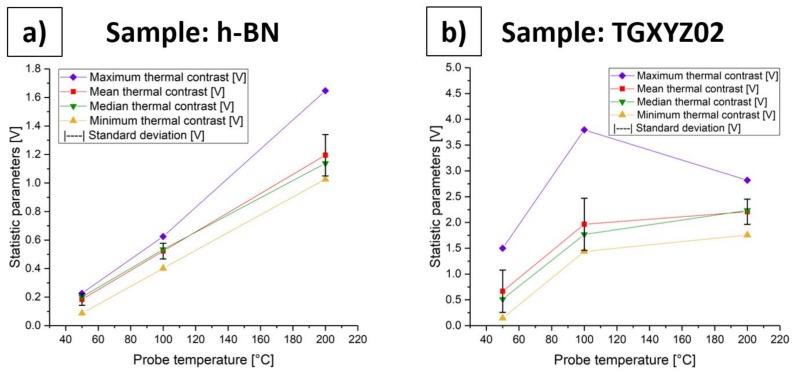
Statistic parameters of Table 2 and Table 3 of both measurements in dependency of the probe temperature. (**a**) Statistic parameters of measurement with h-BN sample; (**b**) Statistic parameters of measurement with TGXYZ02 sample.

Figure 5 illustrates the topography and thermal data of the investigated h-BN sample and Table 2 contains the statistic parameters, which represent the thermal contrast, at the corresponding probe temperatures. The statistic parameters presented in Table 2 are compared to the ones of Table 3 in Figure 7. The step height along the white horizontal cut line in Figure 5a can be estimated to approximately 15.5 nm (mean value of black curve in Figure 5f from 2.09 µm–3.203 µm; n: 58).

The results show that, for the h-BN sample, in this investigation (ultrathin sample with up to 20 nm in thickness with a high in-plane thermal conductivity up to 2000 Wm^−1^ K^−1^), the thermal contrast strongly depends on the probe temperature. As can be seen in Table 2, with an increasing probe temperature, all of the statistic thermal parameters increase (within nearly constant statistic areas). The fact that the thermal signal is significantly higher on top of the step than beyond, leads to the conclusion that the thermal conductivity might be lower on top of the step, when compared to positions beyond. This seems logic, as the step contains more layers of h-BN, which should lead to a decreasing thermal conductivity.

At the edges of the step in Figure 5e super elevations of the thermal signal are visible, predominately for $T_{\mathrm{probe}}=$ 200 °C. These local maxima, which enclose the statistical area (Table 2), are indicators of the topography influences on the thermal signal. The thermal signal should also be constant in contrast to the super elevations, as the layer thickness of h-BN on top of the step is constant. As a result, these values might represent artificially overrated thermal signals. This is a reason why we do not only consider the mean thermal contrast, but also the median thermal contrast in this discussion.

In Figure 5e, it can be observed that the thermal signal right to the step is significantly higher than left to the step and increases gradually. Comparing the corresponding mean values (which in the presented accuracy range do not differ from the median values as in these areas no super elevations occur) of the thermal signals left and right to the step this is confirmed. Right to the step the values are higher by 0.2 V ($T_{\mathrm{probe}}=$ 50 °C), 0.3 V ($T_{\mathrm{probe}}=$ 100 °C), and 0.4 V ($T_{\mathrm{probe}}=$ 200 °C). It seems that in the present measurements the increase of the thermal signal after the step is an artefact that represents a seemingly lower thermal conductivity. In theory, the values left and right of the step should be nearly constant and equal, as the number of h-BN layers is equal left and right of the step. We expect the sample construction to be the reason for this effect. On the one hand, it might be possible that the thermal resistance between h-BN and the SiO_2_ substrate is significantly higher on the right side of the step, being caused by the manufacturing process. As the h-BN thickness right and left of the step is estimated to 6.075 nm ± 1.125 nm, the thermal resistance might have a huge influence on the amount of heat flowing from tip to sample, which would affect the representation of the local thermal conductivities. On the other hand, it could also be possible that, as a matter of fact, the local thermal conductivities on the right side of the step are lower when compared to the left side, also caused by the manufacturing process. These possible reasons need to be considered in more detail in future studies.

It can also be observed (Figure 5f) that both the topography signal and the thermal signals show a kind of ripple especially on the top of the step. In theory, the signals should be almost constant as the layer thickness is nearly constant because of the same number of h-BN layers on and beyond the step. This fact could allow for conclusions according to the oscillation frequency of the entire measuring system (piezo, probe, and sample). When the tip approximates the step, the piezo needs to adjust the z-position of the probe. This leads to varying thermal contact areas between tip and sample, as the force between the tip and sample needs to be adjusted. We can roughly estimate this frequency to 6.7 Hz as a first approach with a tip velocity of approximately 10 µms^−1^ (standard in all our measurements) and an oscillating period of 1.5 µm (estimated by the yellow curve in Figure 5e). This rough approach does not cover the influence of the scanning parameters (proportional and integral gain), the material parameters (Young’s modulus and surface hardness), and the delay of the thermal signal from probe to thermal system. Another impact on this oscillation frequency is the inertia of the thermal system to adjust the probe’s temperature and the thermal resonance frequency of the entire system. All those influences would have to be considered in a next step to explain this ripple in more detail. 

We made investigations with the raised circles of TGXYZ02 (quadrant 1 in Figure 2) under similar conditions in order to compare the statistic parameters and the results of the h-BN measurement to a sample with well-known topography and thermal properties. Figure 6 illustrates the corresponding topography and thermal data and Table 3 contains the statistic parameters, which represent the thermal contrast, at the corresponding probe temperatures. The step height along the white horizontal cut line in Figure 6a can be estimated to approximately 120.0 nm (mean value of black curve in Figure 6f from 1.133 µm–3.984 µm; n: 74).

The measurements with TGXYZ02 lead to similar results according to the thermal contrast when compared to the h-BN sample until a probe temperature of 100 °C. From probe temperature 100 °C up to 200 °C a kind of saturation of the thermal contrast can be found here in contrast to the measurements with the h-BN sample. This fact is further discussed below.

All of the curves in Figure 6e show super elevations at the edges of the step, which represent the local maxima and define the statistic area. These super elevations are also an indicator of the topography influences. It seems that vertical steps extraordinary raise the thermal signal. As a result, these values might represent artificially overrated thermal signals.

In Figure 6e, we can also find a kind of ripple in the topography and the thermal signal. With a tip velocity of approximately 10 µms^−1^ (standard in all of our measurements) and an oscillating period of 1.5 µm (estimated by the blue curve in Figure 6f), we can roughly estimate the frequency of the thermal signal to 6.7 Hz. The oscillation frequency of the topography signal seems to be lower. Here, with an oscillating period of approximately 2 µm (estimated by the black curve in Figure 6f), we come to an oscillation frequency of 5 Hz. It can also be found an x-offset of the thermal signal when compared to the topography signal of −0.118 µm ($T_{\mathrm{probe}}=$ 50 °C), −0.040 µm ($T_{\mathrm{probe}}=$ 100 °C) and +0.156 µm ($T_{\mathrm{probe}}=$ 200 °C) (approximated on the left side of the step).

Figure 7 illustrates the statistic parameters of Table 2 and Table 3 of both measurements. All statistic parameters for the measurement with the h-BN sample increase with an increasing probe temperature. Mean, median, and minimum thermal contrast increase more or less linearly, whereas the maximum thermal contrast shows a greater rise starting from probe temperature 100 °C upwards, which also causes a greater standard deviation. The measurement with TGXYZ02 also shows an increase of mean, median, and minimum thermal contrast with an increasing probe temperature, but shows a kind of saturation starting from probe temperature 100 °C upwards. This also becomes obvious by the decreasing maximum thermal contrast and, hence, standard deviation from 100 °C to 200 °C. In summary, we can say that the thermal contrast in our h-BN measurements increases with increasing probe temperature until 200 °C, whereas, in our measurements, with the TGXYZ02 sample, we find indicators of saturation of the thermal contrast at 100 °C. A possible reason for this might be the influence of the high in-plane thermal conductivity of the h-BN sample and its ultra-thin film thickness. Furthermore, for future comparisons, we recommend choosing the median thermal contrast as the comparative parameter instead of the mean thermal contrast in statistic areas, which contain super elevations. The reason for this is that the median thermal contrast is able to compensate the effect of the artificial super elevations. For demonstration purposes, we evaluated both, the median and mean thermal contrast in this work.

### 4.3. Angle Dependency of the SThM Signal

**Figure 8:**
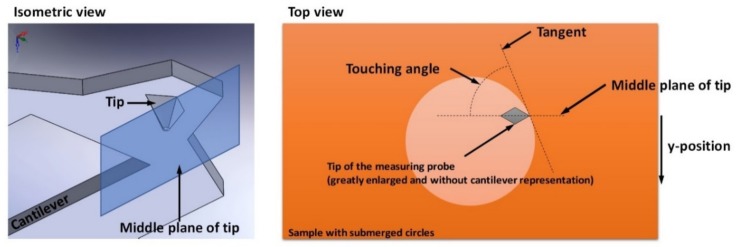
Definition of the touching angle as the angle between the middle plane of the tip and the tangent to the submerged circle structure.

**Figure 9:**
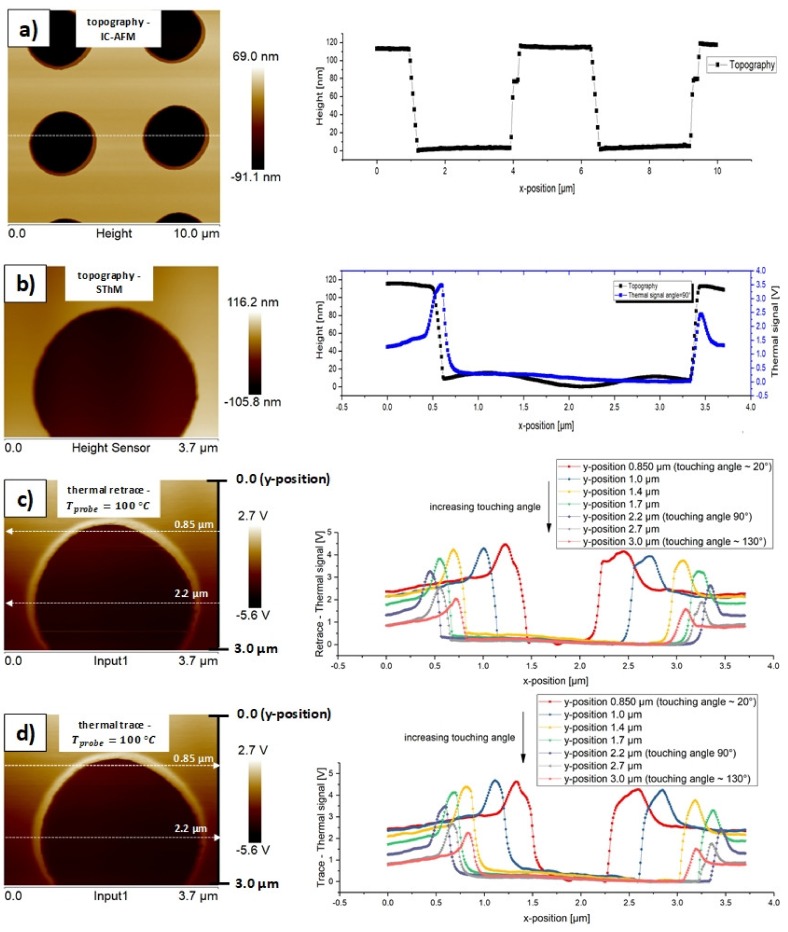
(**a**) Topography image of the submerged circles of the test sample TGXYZ02 (quadrant 3 in Figure 2) with the corresponding two-dimensional (2D) plot along the white cut line performed using intermittent-contact mode (IC-AFM); (**b**) Unmodified topography image of the submerged circles of the test sample TGXYZ02 (quadrant 3 in Figure 2) with the corresponding 2D plot along the white cut line performed using scanning thermal microscopy (SThM); (**c**) Unmodified thermal image under a probe temperature of 100 °C with the corresponding 2D plots of retrace under different y-positions of the cut line and hence touching angles; (**d**) Unmodified thermal image under a probe temperature of 100 °C with the corresponding 2D plots of trace under different y-positions of the cut line and hence touching angles.

In a further step, we investigated the angle dependency of the thermal signal. The investigation was performed while using the submerged circles of TGXYZ02 (quadrant 3 in Figure 2) as a circle structure of this kind is suitable for illustrating thermal signals with different touching angles in a single thermal image. Figure 8 represents the definition of the touching angle. Figure 9a,b illustrate the topography of the submerged circles of the test sample TGXYZ02 (quadrant 3 in Figure 2). The following definitions were made:

- Touching angle: Angle between the tangent of the step and the middle plane of the tip, looking upside down according to Figure 8. We vary the touching angle by moving the middle plane of the tip by varying the y-positions of the cut lines within the software. The touching angle also increases with increasing y-position. The 90° case is the case of symmetry.
- Thermal contrast: Maximum value of the thermal signal for each curve in Figure 9c,d. The minimum value was set to zero, which is permitted, because the thermal signal might be interpreted as a potential represented by voltage.
- Absolute strength of super elevations: Highest value of each super elevation subtracted by the minimum value in the nearly constant area right of the super elevations. This evaluation was performed for all of the super elevations on the right side in Figure 9c,d.
- Relative strength of super elevations: Absolute strength of each super elevation divided by the minimum value in the nearly constant area right of the super elevations in Figure 9c,d. This evaluation was performed for all of the super elevations on the right side in Figure 9c,d.

Figure 9a was recorded by IC-AFM to overcome disadvantages of the SThM topography image (Figure 9b). As the IC-AFM tip is much sharper as the SThM tip, it displays the topography with more precision than SThM. In addition, a 1st order plane fit with the NanoScope software corrected the horizontal sample inclination. The depth of the submerged circles can be measured to 114.7 nm (mean value of black curve in Figure 9a between 4.219 µm and 6.289 µm; n: 54). Figure 9c,d show the retrace and trace curves for different y-position of the horizontal cut line and, thus, different touching angles. Table 4 contains with the y-position associated statistic parameters of the thermal contrast.

**Figure 10:**
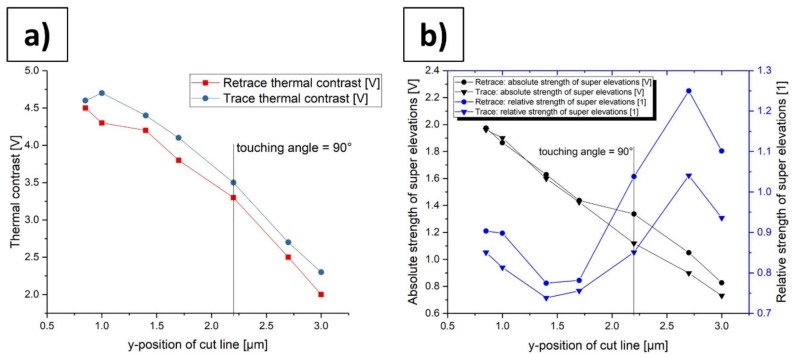
Statistic evaluation of the thermal contrast and the strength of the super elevations. (**a**) Comparison of the thermal contrast of trace and retrace in dependency of the y-position of the cut line and hence the touching angle; (**b**) Comparison of the absolute strength of the super elevations of trace and retrace (black curves) and comparison of the relative strength of the super elevations of trace and retrace (blue curves) in dependency of the y-position of the cut line and hence the touching angle.

In Table 4, it might be observed that with increasing y-position of the cut line the thermal contrast for retrace and trace decreases (with one exception at y-position 1.0 µm). This indicates that the thermal contrast does not depend on the touching angle. If the thermal signal would depend on the touching angle, the thermal contrast would rise after passing the symmetric angle of 90° (y-position 2.2 µm). A possible reason for this decrease in thermal contrast is a progressive damage and pollution of the tip with increasing y-position, which would affect the effective thermal contact area between tip and sample and influence the thermal contrast. This effect needs to be investigated in more detail in future studies by using new probes, different materials, and larger dimensions of the investigated areas. It might also be possible to investigate this effect by using an SThM system included in an SEM system, which makes it possible to get “live-videos” of the scanning process. It can also be seen from Table 4 that the absolute and relative strength of the super elevations of retrace is always greater than for trace (with one exception at y-position 1.0 µm). Furthermore, it can be stated that with increasing y-position of the cut line the absolute strength for retrace and trace decreases. The course of the relative strength for both retrace and trace is not axisymmetric to the 90° line. These observations, which can also be seen in Figure 10b, are indicators that the absolute and relative strength of the super elevations do not depend on the touching angle. Figure 10a shows the thermal contrasts of trace and retrace over the y-position of the cut line and, hence, of the touching angle. The thermal contrast value decreases with an increasing y-position of the cut line and it is always greater in the trace direction than in the corresponding retrace direction. Figure 10b shows the absolute and relative strength of the super elevations of trace and retrace over the y-position of the cut line and, hence, of the touching angle. All four curves in Figure 10b do not show signs for axis symmetry to the 90° line. These results appear somehow surprising, because in theory we would expect a small dependency of the thermal contrast and the strength of the super elevations on the touching angle as the touching areas vary with the touching angle. Obviously, this effect is negligible, at least for the present investigation. 

In Figure 9c,d, we can also see that the thermal signals slightly decrease at the bottom of the step for each probe temperature with an increasing x-position for all curves. The largest difference observable is the mirroring of the sharp and smooth transition zones at the bottom left and right of each curve. In trace the sharp zones are on the bottom right, whereas in retrace they are on the bottom left. The super elevations, which partly represent the thermal contrast here, are another strong indicator of the topography influence on the thermal signal. In each measurement, they occur exactly at the beginning and end of the step where the thermal contact areas vary biggest. We can not find a ripple in the thermal signals whereas the topography signal shows one, which is supposed to be artificial. With a tip velocity of approximately 10 µms^−1^ (standard in all our measurements) and an oscillating period of 1.95 µm (estimated by the black curve in Figure 9b), we can roughly estimate the frequency expected for the thermal signal to be 5.1 Hz. We do not find a strong indicator that the mean thermal signals are significantly different when comparing the values left and right of the step for each curve. This seems logic as the local thermal conductivities left and right of the step should be equal as material and layer thickness are the same here. The thermal conductivity at the bottom of the step must be higher as the material is bulk Si here, which has a higher thermal conductivity (bulk Si: 163 Wm^−1^ K^−1^) than SiO_2_ beyond (bulk SiO_2_: 1.38 Wm^−1^ K^−1^). Measurement and theory match in this point.

## 5. Conclusions

In this work, we applied SThM on diverse thin and ultrathin films to approach and explore limitations of this method. Several conclusions can be drawn:

1. Thermal signals at vertical steps (especially occurring super elevations at the beginning and end of a vertical step) always have to be questioned critically as the thermal signal may not represent the correct local thermal conductivity here. We suggest ignoring the thermal signal near the vertical steps. The step heights in the present investigations varied from 15.5 nm (h-BN sample) to 120.0 nm (TGXYZ02 sample). In some measurements we also found that after passing a step the thermal signal is significantly greater than before, where it should be equal (h-BN sample: 0.2 V ($T_{\mathrm{probe}}=$ 50 °C), 0.3 V ($T_{\mathrm{probe}}=$ 100 °C), and 0.4 V ($T_{\mathrm{probe}}=$ 200 °C)). If present, this effect also has to be critically considered.
2. SThM is a useful technique that is applied to ultra-flat surfaces but results according to samples with a high roughness (CuI sample had an R_q_ of 110 nm, whereas the h-BN sample had an R_q_ of 4.15 nm) must always be questioned critically. Samples with a super-flat surface without vertical steps are especially suitable for SThM technique. Between step angels of 90° and flat samples, in our current investigations, we were not able to estimate or deduce some kind of critical step angle with respect to the influence on the thermal signal. Further research needs to be done.
3. The combination of low film thickness (<50 nm), high surface roughness (R_q_ > 50 nm), vertical steps, and high in-plane thermal conductivity (>200 Wm^−1^ K^−1^) makes the results more unsatisfying and more difficult. These are the main limiting factors of useful SThM measurements.
4. Our investigations show that the thermal contrast depends on the probe temperature. This was predominantly the case for the ultrathin h-BN sample (ultrathin sample with a film thickness at or below 23 nm with a high in-plane thermal conductivity up to 2000 Wm^−1^ K^−1^). Therefore, for similar h-BN samples, we suggest a probe temperature of 200 °C and for samples similar to TGXYZ02 (step height 120.0 nm consisting of Si and SiO_2_) we suggest probe temperatures of 100 °C. Greater probe temperatures may also increase the thermal contrast, but lead to a higher mechanical damage of tip and sample. For other samples, researchers need to be aware of possible saturation effects and it might be necessary to find some kind of optimal probe temperature, depending on the sample.
5. There is no indicator that the thermal contrast depends on the touching angle. In theory, we would expect a small dependency of the thermal contrast on the touching angle, as the touching areas vary depending on the touching angle. Our investigation with TGXYZ02 shows that this effect obviously is negligible. The thermal contrast decreased from 4.5 V to 2.0 V (retrace curves of TGXYZ02, submerged circles) and from 4.6 V to 2.3 V (trace curves of TGXYZ02, submerged circles) with an increasing touching angle, but did not show signs of axis symmetry to the 90° line. Additionally, the absolute and relative strength of super elevations did not depend on the touching angle. The relative strength of the super elevations varied from 0.77 to 1.25 (retrace curves of TGXYZ02, submerged circles) and from 0.74 to 1.04 (trace curves of TGXYZ02, submerged circles) and did not show signs of axis symmetry to the 90° line.
6. Some of our thermal and topography signals showed a kind of ripple after passing a vertical step. The IC-AFM measurements confirmed that the ripples in topography are artefacts and, hence, we also suppose the ripple in the thermal signals to be artificial. The frequency of those ripples can be estimated between 5 Hz (topography signal of TGXYZ02) and 6.7 Hz (thermal signal of TGXYZ02). We suppose the natural frequency of the cantilever, the surface hardness of the sample, and the proportional gain to influence this ripple most but unfortunately, we were not able to explain the origin of the ripples sufficiently and, thus, further research is necessary.
7. The course of the thermal signal of the h-BN sample suggests the thermal conductivity to decrease with increasing film thickness in our investigations. Absolute numbers of the local thermal conductivities cannot be obtained with the SThM system used in this work.
8. Last but not least, we were able to successfully apply the SThM method to h-BN films at or below 23 nm, which has not been reported before (general h-BN thickness: 6.075 nm ± 1.125 nm; h-BN thickness on top of the vertical step: 21.575 nm ± 1.125 nm).

The achieved results demonstrate that the limits of the method to qualitatively map the thermal conductivity of ultrathin films are not reached yet. Further research is necessary in order to fully understand the capabilities and limitations of SThM. Especially with respect to the interdependence of topography and the thermal signal it is necessary to increase the number of SThM measurements and to further explore different materials, such as diamond like films [25,26], surface properties, step angles, and scanning parameters, such as the tip velocity. By this means and in combination with FEM simulations it could be possible to reduce or even eliminate the topography influence in future thermal images.

## Figures and Tables

**Table 1:** Probe Calibration points in this work.

**Temperature (°C)**	55 ($T_{m}$ of PCL)	116 ($T_{m}$ of PE)	235 ($T_{m}$ of PET)
**Heating voltage (V)**	2.90 ± 0.03	4.80 ± 0.03	6.78 ± 0.03

**Table 2:** Investigation of the thermal contrast (curves in Figure 5e) at different probe temperatures by means of specific statistic parameters.

Probe Temperature (°C)	Statistic Area (µm–µm)	Mean Thermal Contrast (V)	Standard Deviation (V)	Minimum Thermal Contrast (V)	Median Thermal Contrast (V)	Maximum Thermal Contrast (V)
50	2.070–3.203 (n = 59)	0.184	0.041	0.087	0.202	0.227
100	2.070–3.203 (n = 59)	0.523	0.055	0.402	0.534	0.625
200	2.090–3.320 (n = 64)	1.195	0.145	1.027	1.138	1.647

**Table 3:** Investigation of the thermal contrast (curves in Figure 6e) at different probe temperatures by means of specific statistic parameters.

Probe Temperature (°C)	Statistic Area (µm–µm)	Mean Thermal Contrast (V)	Standard Deviation (V)	Minimum Thermal Contrast (V)	Median Thermal Contrast (V)	Maximum Thermal Contrast (V)
50	1.094–4.023 (n = 76)	0.667	0.410	0.145	0.512	1.499
100	1.094–4.063 (n = 77)	1.964	0.505	1.438	1.768	3.793
200	1.289–4.180 (n = 75)	2.208	0.246	1.756	2.235	2.819

**Table 4:** Thermal contrast, and absolute and relative strength of the super elevations of retrace and trace curves in Figure 9c,d at different y-positions of the cut line and hence different touching angles.

**y-Position of Cut Line (µm)**	0.85	1.0	1.4	1.7	2.2 (angle = 90°)	2.7	3.0
**Retrace: Thermal Contrast (V)**	4.5	4.3	4.2	3.8	3.3	2.5	2.0
**Trace: Thermal Contrast (V)**	4.6	4.7	4.4	4.1	3.5	2.7	2.3
**Retrace: Absolute Strength of Super Elevations (V)**	1.97	1.87	1.63	1.44	1.34	1.05	0.83
**Trace: Absolute Strength of Super Elevations (V)**	1.96	1.90	1.60	1.43	1.12	0.90	0.73
**Retrace: Relative Strength of Super Elevations (1)**	0.90	0.90	0.77	0.78	1.04	1.25	1.10
**Trace: Relative Strength of Super Elevations (1)**	0.85	0.81	0.74	0.76	0.85	1.04	0.94
